# Clinical and radiological outcome after anterior cruciate ligament reconstruction using the T-lock Osteotrans resorbable tendon anchor: early experience and midterm follow-up

**DOI:** 10.1186/s12891-020-03863-5

**Published:** 2020-12-18

**Authors:** Y. Bangert, A. Jaber, F. Wünnemann, G. Berrsche, N. Streich, C. Rehnitz, H. Ott, A. Barié

**Affiliations:** 1 Department of Orthopaedic and Trauma Surgery, Center for Orthopaedics, Trauma Surgery and Spinal Cord Injury, Heidelberg University Hospital, Schlierbacher Landstrasse 200a, 69118 Heidelberg, Germany; 2 Department of diagnostic and interventional radiology, Heidelberg University Hospital, Schlierbacher Landstrasse 200a, 69118 Heidelberg, Germany; 3 Center for Joint Surgery and Sport injuries, Sportopaedie Heidelberg, Clinic St. Elisabeth Heidelberg, Max-Reger-Straße 5-7, 69121 Heidelberg, Germany; 4 Sporthologicum – Center for Sport and Joint injuries, Siesmayerstraße 44, 60323 Frankfurt am Main, Germany

**Keywords:** Anterior cruciate ligament reconstruction, T-lock, Femoral fixation, Athletes, Arthroscopy, Hamstring

## Abstract

**Purpose:**

Reconstruction of the Anterior cruciate ligament (ACL) using tendon grafting is an established method for restoring knee function and stability. Multiple methods are established for graft fixation. Several involve anchoring the autograft distant to the joint with hardware that remains implanted. This study reports the first early to midterm results in patients who received ACL reconstruction (ACLR) using the T-Lock Osteotrans femoral near joint fixation method with a tibial fixation using the BioactIF Osteotrans interference screw.

**Methods:**

This consecutive prospective series included 20 Patients (14 Male, 6 Female) with a primary ACL rupture. All patients were treated with an ACLR using a semitendinosus autograft fixated with the T-Lock Osteotrans and were followed-up postoperatively. The following parameters were assessed: Side-to-side difference of the posterior-anterior translation measured using the KT-1000 arthrometer, Tegner activity score, Lysholm score, IKDC subjective knee evaluation form. Magnetic resonance imaging (MRI) was done to assess tunnel enlargement and integrity of the anchoring device.

**Results:**

The average follow-up duration was 2 years (range 1–4.2 years). One patient was lost to follow-up. Two Patients suffered a traumatic ACL re-rupture 2 years postoperatively and received a 2-stage revision ACLR. Difference in the posterior-anterior translation was 1.8 mm (range 0–5). The median Tegner score was 6 (range 4–10) and 9 patients (45%) returned to their preinjury level of activity. The mean IKDC subjective knee evaluation form scored 91 points (range 77–100). The mean Lysholm score was 86 points (74–96). All mentioned scores were significantly better compared to preoperative values. No relevant tunnel enlargement was seen on MRI. The anchoring device was evaluated to be intact in all patients.

**Conclusion:**

ACLR with the aforementioned procedure leads to good clinical and radiological outcome.

## Background

The anterior cruciate ligament (ACL) is an important stabilizing ligament of the knee and is frequently injured by athletes. There are between 100,000 and 200,000 ACL ruptures per year in the United States. Direct and indirect costs constitute to more than $7 billion annually [13]. In Germany, about 50,000 ACL ruptures occur each year [29]. ACL injuries can be functionally disabling. They also predispose patients to further damage as well as early onset of degenerative changes [12].

ACL reconstruction (ACLR) restores mainly the anterior–posterior knee stability after injury [19]. This is typically achieved by replacing the original ACL with an autograft tendon [24]. The graft can be fixed in various ways. Essentially, a distinction is made between anchoring the graft close to and distant from the joint. During fixation close to the joint, the graft is anchored near the original insertion of the ACL. This can be achieved using different methods (mostly interference screws, cross-pins, staples or a prepared bone block using the press-fit technique without implants). The interference screws are divided into metal and biodegradable interference screws. Both of which offer adequate graft fixation [6]. However, when compared to the metal interference screws, biodegradable interference screws offer an undisturbed imaging diagnosis, a higher tear resistance and an easier access during revision operations [18, 22, 31]. Disadvantages of graft fixation close to the joint have so far been the risk of the graft twisting when the screw is being inserted, anchoring problems with low bone density and the need to remove the metal interference screws during revision operations. In addition, when using screws for femoral anchoring, the graft is displaced toward the edge of the borehole. The insertion area of ​​the femoral transplant is thereby reduced, and the footprint is therefore not anatomically reconstructed.

In the case of graft fixation distant from the joint, a four-hole titanium plate otherwise known as “Endobutton” or “Suture Plate” is used proximally and a titanium button or “Suture Disc” is used distally. Disadvantages of the graft fixation distant from the joint are shear forces between the graft and the bone tunnel due to the bungee effect and a certain freedom of movement of the graft in the drill channel. As a result of the shear forces and the freedom of movement, there may be a delay in healing of the graft and an expansion of the drill channel, also known a tunnel widening [11, 21].

Despite the availability of several femoral anchoring systems, none were able to establish themselves as a new standard procedure. The placement of the femoral drill channel and the exact anatomical reconstruction of the insertion area of the original ACL are currently the subject of clinical research, since they seem to be largely responsible for the success of a ACL reconstruction and the prevention of transplant failure [20].

The T-Lock Osteotrans tendon anchor is a bioactive and bioabsorbable tendon anchor for near joint femoral fixation. The BioactIF Osteotrans is a bioabsorbable interference screw. These products market tendon fixation near the joint, as well as longterm bioabsorbability and osteoconductivity that leads to good osseous healing postoperatively. To the authors’ knowledge, no studies exist that report the outcome and reliability of this technique. This study demonstrates the first early to midterm results for this fixation method. The semitendinosus autograft was chosen for this procedure since it was recommended by the product manufacturer. It also has good long-term outcome and a relatively low donor site morbidity [9, 28].

## Methods

**Fig. 1:**
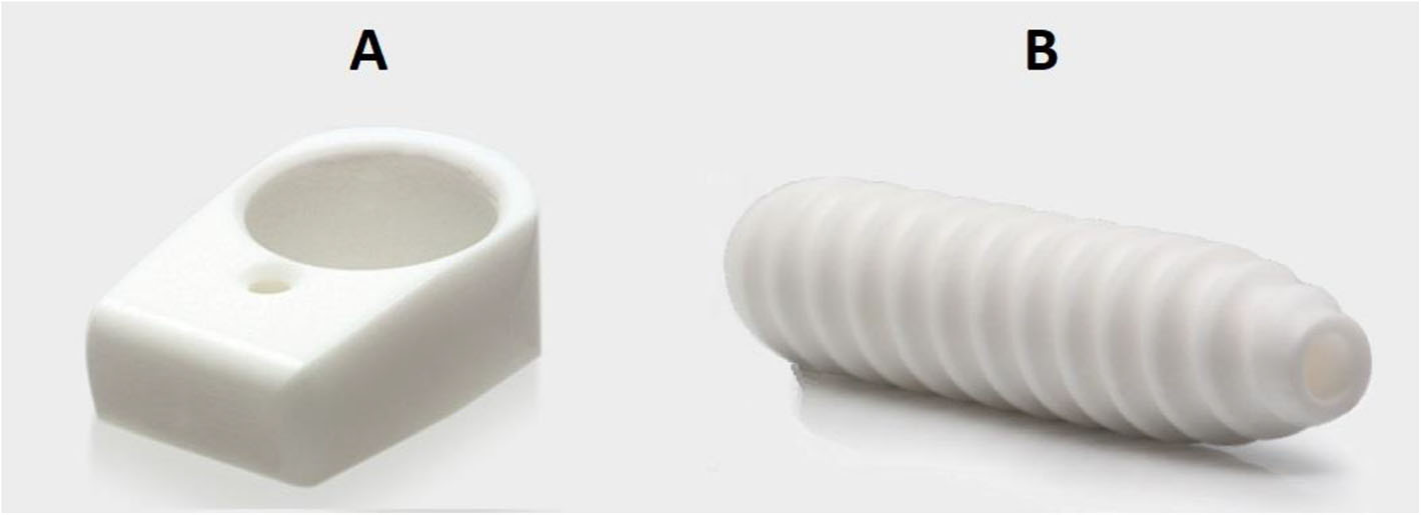
**a** T-Lock Osteotrans. **b** BioactIF Osteotrans interference screw

**Fig. 2:**
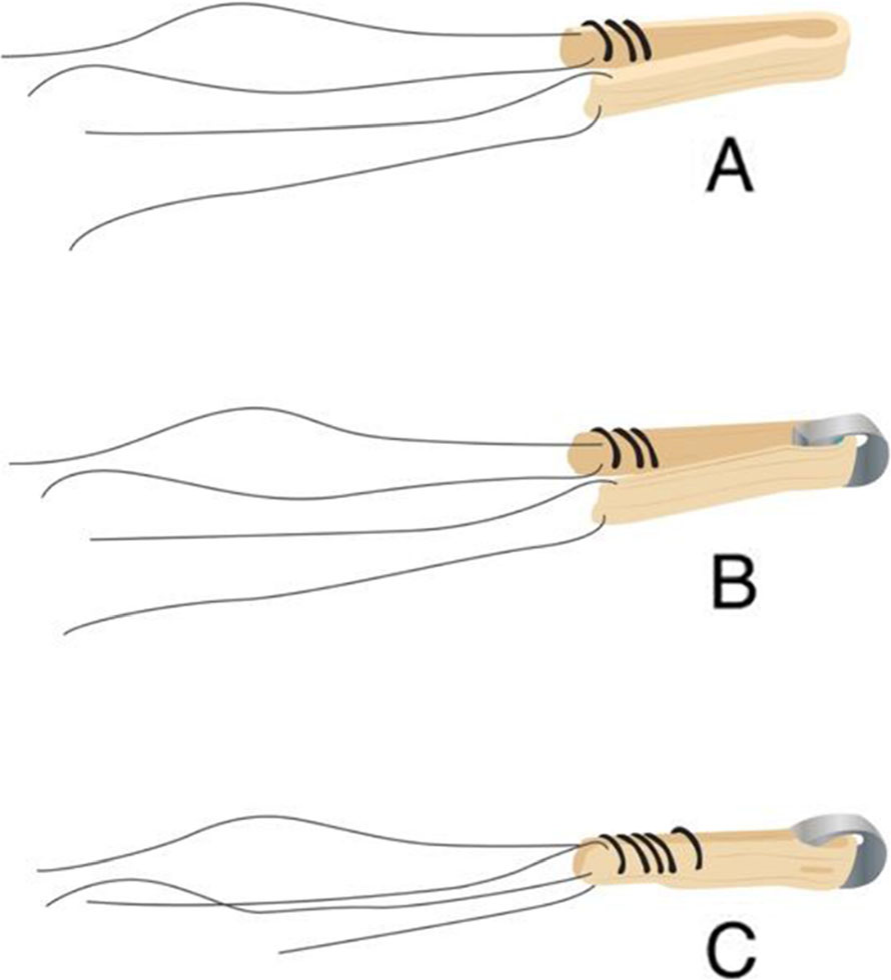
**a** Removal of semitendinosus tendon, removal of residual muscle tissue and joining of the two ends of the tendon. Suturing with nonresorbable strength 2 sutures over a length of approximately 20 mm. Placing a stay-suture in the tendon loop at the other end of the double tendon and determining the diameter of the 4-way transplant. **b** Selection of the T-lock in accordance with the determined transplant diameter and passing the transplant through the large hole of the implant. A four-way transplant is created. **c** Fixation of the tendon loop through the small hole of the implant with nonresorbable fibers (polyester strength 2). Suturing the distal end of the transplant with resorbable threads

**Fig. 3:**
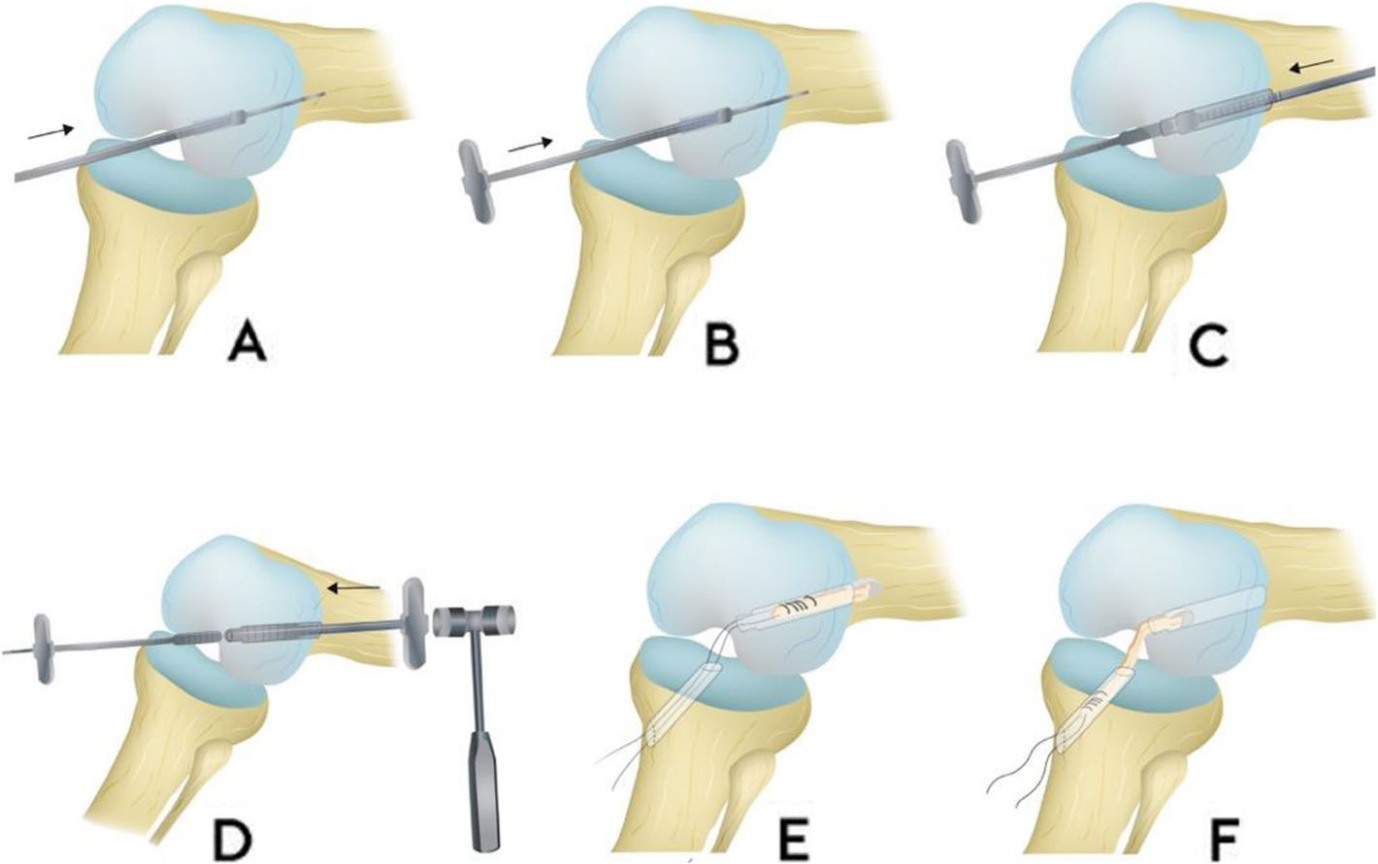
**a** Inside-out drilling: Positioning of the target drill wire with suitable target instrument through the anteromedial portal. Over drilling with cannulated drill. Drill diameter is the same as the transplant diameter. **b** Positioning of the impactor: Positioning of the cannulated impactor of the same diameter 10 m deep in the femoral channel. Stab incision via the femorally drilled target drill. **c** Outside-in drilling: Drilling using a cannulated drill via the target drill wire until the impactor is reached. **d** Impacting the drill channel: Impacting a stage impactor from outside via the drill hole wire until the top of the stage impactor is visible in the joint. The impactor positioned in the drill channel is knocked back in this process. **e** Drawing the transplant into the femoral channel from proximally to distally until the T-Lock Osteotrans tendon anchor is pressed in close to the joint. **f** Conditioning of the transplant by moving the knee many times from maximum extension to flexion with vigorous distal traction

This was a prospective consecutive series which included 20 patients (14 Male, 6 Female) who sustained a primary rupture of the ACL. All patients received an ACLR using the semitendinosus autograft fixated using the T-Lock Osteotrans for the femoral fixation and BioactIF Osteotrans interference screw for tibial fixation (Fig. 1). The preparation of the autograft and femoral fixation are demonstrated in Figs. 2 and 3. Eight patients also suffered from concomitant meniscal damage (6 medial meniscus, 2 lateral meniscus). Of these, 5 patients received a meniscal refixation and 3 patients received a partial meniscectomy. The mean age at the time of surgery was 25.6 years (Range 17–44). Ethical clearance was acquired from the institutional ethical committee with the reference number S-488. All patients signed a written agreement to be included in this study.

The inclusion criteria comprised of the following points: Primary ACL rupture, preinjury Tegner score ≥ 4, skeletal maturity, < 45 years of age at the time of surgery. Exclusion criteria included active infection, bone fractures, injuries of the lateral collateral ligament and the posterior cruciate ligament, history of prior knee surgery and presence of chondromalacia higher than grade II according to Outerbridge [26].

During the preoperative examination, anterior sagittal laxity was objectively assessed by means of instrumented measurement using the KT-1000™ Knee Ligament Arthrometer® manufactured by MEDmetric® Corporation (http://www.medicalproductguide.com/companies/1364/medmetric_corp, MEDmetric® Corporation, 7542 Trade Street, San Diego, CA 92121; Patent no. 4,583,555) and a tension force of 134 N [1]. This was also examined on follow-up and compared to the contralateral side. Additionally, the following scores were assessed preoperatively and on follow-up: Tegner score, Lysholm score and IKDC subjective knee evaluation form [3, 10, 30]. Tegner score preinjury was also retrieved.

Postoperatively, all participants underwent standardized rehabilitation regimens. The postoperative rehabilitation program involved range of motion re-acquisition with full extension in the first 2 weeks. Prone hangs and bridging exercises were considered. Progressive weight-bearing assisted by crutches was performed. Individuals were advised to avoid open-chain exercises and extension against resistance for the first 6 months. These postoperative rehabilitation regimens were uniform for all patients and were at times, individually tweaked as required. Such as in the case of accompanying meniscal damage where patients received a meniscal refixation. Following this procedure, a brace was used with gradual limitation of knee flexion for a period of 6 weeks (gradual increase of 30 degrees every 2 weeks).

Besides a conventional AP-Radiograph of the knee, the radiological evaluation also included Magnetic Resonance Imaging (MRI). For all patients, an MRI was planned 1 and 3 years postoperatively. In total, 28 MRIs were done. Of these, 26 MRIs were conducted in the department of diagnostic and interventional radiology in the same institute. The remaining two examinations were done in outpatient facilities. The 26 examinations were facilitated using a 70-cm open bore 3-T MR Scanner (Magnetom Verio, Siemens Healthineers, Erlangen, Germany) with an 18-channel total imaging matrix and a dedicated knee-coil. The patients were positioned feet-first and supine with the knee in a neutral position. The knee was positioned as close as possible to the isocenter of the magnet. In order to minimize movement artifacts, radiographers were advised to stabilize the knee joint. For morphologic imaging assessment of the knee, the in-house standard knee MRI protocol was used. It included proton-density weighted fat saturated sequences in all three imaging planes as well as a sagittal T1weighted sequence, a coronal proton-density weighted sequence and a sagittal 3D DESS-Sequence that was reformatted in all three planes.

All studies were evaluated by a musculoskeletal radiologist with 5 years of experience in musculoskeletal MRI. The radiologist determined the slice selection, magnification and windowing parameters. The Ambient light was kept at minimum during the reading sessions. Femoral tunnel enlargement was measured by subdividing the femoral tunnel into a proximal, mid and distal section on the coronal proton-density, coronal proton-density fat-saturated or coronal T1-weighted images. The diameter was measured perpendicular to the tunnel border. In patients with two MRI follow-up examinations the tunnel diameter was measured in similar positions in order to enable the assessment of a tunnel enlargement. The T-Lock anchoring device was evaluated using a 4-point classification system according to Cossey et al. and classified as intact, deformed, fractured or not visible [5]. In accordance to Figueroa et al., the graft signal was evaluated with regards to the predominating signal intensity [7]. The bone-graft interface was evaluated with regard to the presence of a fluid-intense rim surrounding the graft within in the tunnel using a 3-point scale (no fluid signal, < 50% fluid signal and > 50% fluid signal). Furthermore, Bone-Marrow-Edema (BME) was classified binarily as either absent or present [27].

The statistical analysis was performed using SPSS version 26. A *p*-value of 0.05 significance level. The Wilcoxon test as a paired non-parametric test was performed to compare pre- und postoperative parameters.

Data acquisition and analysis were performed in compliance with protocols approved by the Ethical Committee of the medical faculty of the Ruprecht-Karls-University Heidelberg (Reference number: S-488). The study was registered in the German Register of Clinical Studies and was conducted in accordance with the Declaration of Helsinki. All Patients gave written consent to participate in the study.

## Results

All patients received a thorough examination before the ACLR. The median Tegner score was 7 (range 4–10) preinjury and 3 (range 0–6) preoperatively. The mean Lysholm score was 70 points (range 53–95) and the mean IKDC score was 59.7 points (range 42.5–79.3) preoperatively. The side-to-side difference of the posterior-anterior translation measured using the KT-1000 Arthrometer yielded a mean value of 5.1 mm (range 1–8 mm).

The mean follow-up duration was 2 years (range 1–4.2 years). One patient was lost to follow-up. One patient underwent an arthroscopy 10 months postoperatively for a meniscal lesion. Two Patients suffered a traumatic ACL re-rupture 2 years postoperatively. One of these patients underwent a 2-stage ACLR including an initial procedure with a bone graft followed by ACL reconstruction with the quadriceps bone tendon autograft. The other patient received the first stage surgery in the same institute and the 2nd stage surgery in another hospital. The results of the two patients until the re-rupture were included in the results.

On final follow-up, the average side-to-side difference of the posterior-anterior translation measured using the KT-1000 arthrometer was 1.8 mm (range 0 to 5). This increase compared to the preoperative value was statistically significant (*p* < 0.0001). The median Tegner score was 6 (range 4–10) and 9 patients (45%) returned to their preinjury level of activity. The mean IKDC subjective knee evaluation form scored 91 points (range 77–100). The mean Lysholm score was 86 points (74–96). Statistically, significant improvements were found in all functional outcomes on follow-up compared to preoperative data using the Wilcoxon Test; Tegner (*p* < 0.0001), Lysholm Score (*p* < 0.0001) and IKDC score (*p* < 0.0001). All patients were satisfied with the result on follow-up.

### MRI results

**Fig. 4:**
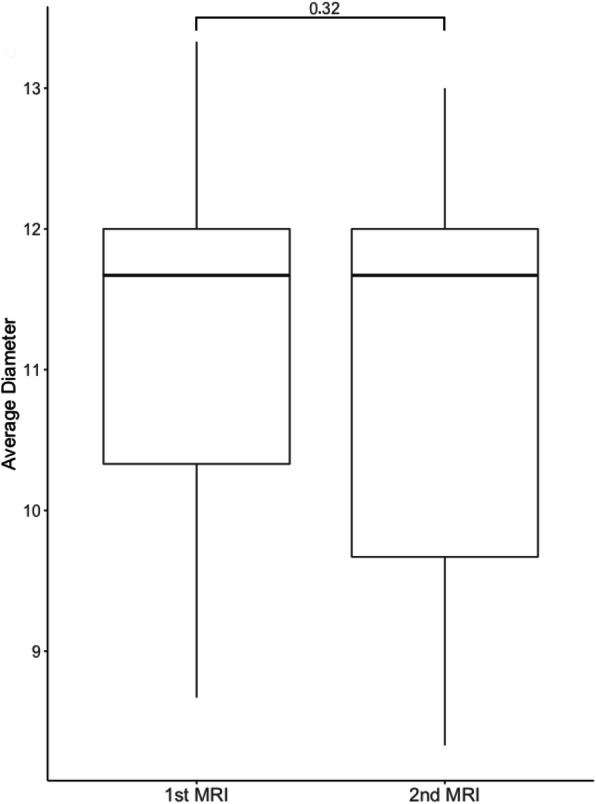
Boxplots of the average femoral tunnel diameter in 9 patients with two follow-up MRI examinations. The mean interval between both MRI examinations was 19.67 ± 6.4 months

The average diameter of the femoral tunnel measured on coronal MRI scans was 9.72 mm in the proximal third of the tunnel, 11.22 mm in the mid portion of the tunnel and 10.94 mm in the distal third resulting in an average overall tunnel diameter 10.63 mm. In the 9 patients with two follow-up MRI scans, there was no significant difference in the tunnel diameter between the first and the second MRI scan (*p* = 0.32) (Fig. 4).

The T-Lock anchoring device was evaluable in 21 of 28 MRI examinations (75%). In seven MRI examinations the anchoring device was obscured by procedure related metal artifacts. These were probably a result of metal debris from the drill. If visible, the anchoring device was classified as intact in 21 of 21 MRI examinations (100%). Thus, there were no cases of deformation or fracturing of the T-Lock-Anchor.

The predominating graft signal was hypointense in 21 out of 28 MRI examinations (75%), isointense in 4 MRI scans (14%) and predominatly hyperintense in one MRI examination (3.5%). In two MRI examinations (7%) the autograft could not be evaluated due to a re-rupture.

With regard to the bone-graft-interface, a femoral fluid-intense signal rim was found in 71% of the MRI examinations and a tibial fluid-intense rim could be depicted in 60% of the imaging studies. However, in the majority of cases the fluid signal covered less than 50% of the bone-graft-interface (80% femoral, 88% tibial).

A BME adjacent to the femoral graft tunnel was found in 13% of the MRI examinations and a tibial BME was diagnosed in 28% of the MRI scans.

## Discussion

ACLR remains one of the most common orthopaedic procedures that are particularly related to sport orthopaedics and traumatology [2]. The technique continues to evolve as the incidence of ACL ruptures is on the rise.

This study reports good clinical and functional outcome for the above-mentioned technique that achieves a near joint femoral fixation of the hamstring autograft as compared to primary results using established methods [23, 25]. The presumable re-rupture rate of 10 is comparable to established techniques in the medical literature [15]. The pullout strength of the replacement ACL construct remains untested. Nevertheless, both patients who suffered a traumatic re-rupture did so during high level sport activities (football and rugby). A total of 9 patients returned to their preinjury level of sport according to Tegner score. However, only one patient reported that competitive sport (football) was not possible anymore because of the operated knee.

Both the T-lock Osteotrans and the BioactIF Osteotrans are made of Poly-L-Lactide (PLLA) and uninterred Hydroxylapatite (uHA). PLA degrades into innocuous lactic acid and is broken down inside the body within 6 months to 2 years [17]. Hydroxylapatite has several medical applications in medicine and dentistry. It proved to have good osseointegration properties but the biodegradation process is slower and would take up to several years [16, 33].

**Fig. 5:**
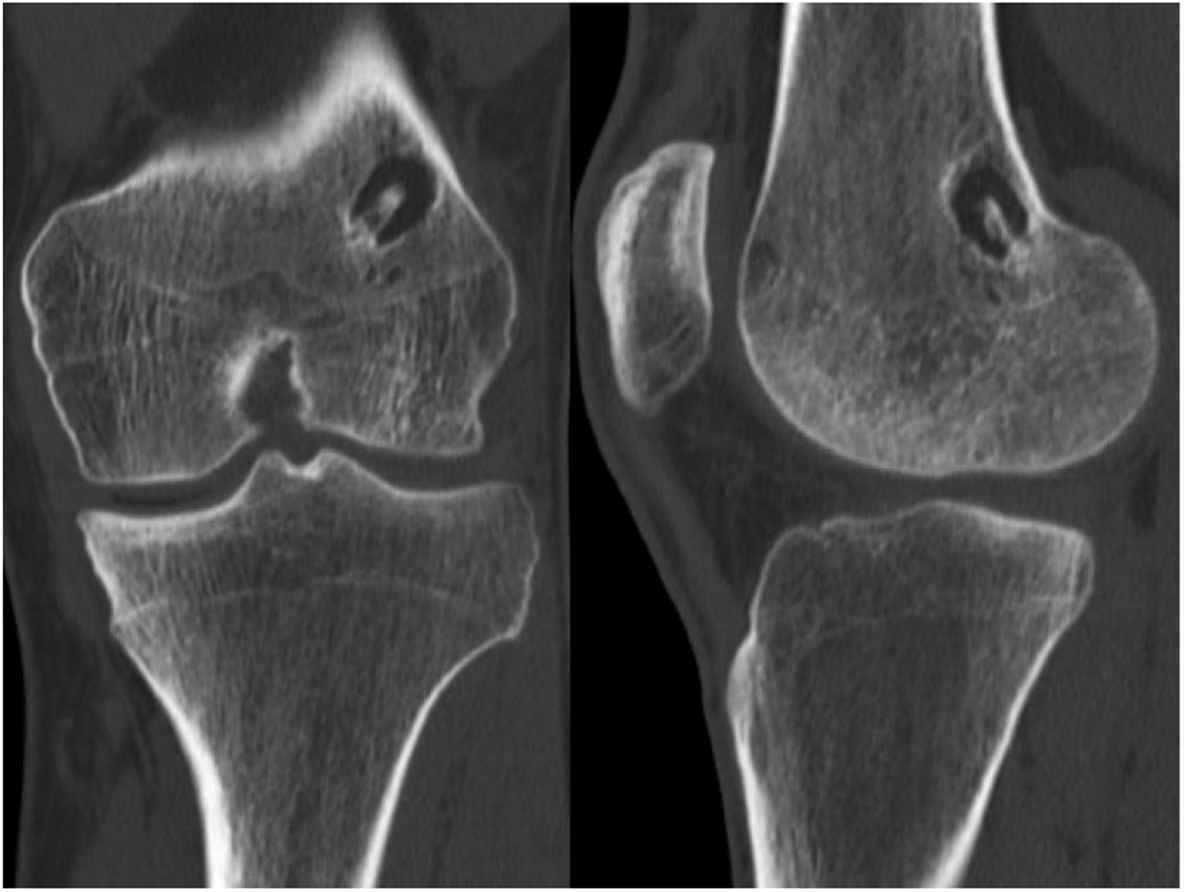
Frontal and sagittal view CT of a 20-year old patient with a re-rupture 2 years after ACL reconstruction. The outer femoral drill canal measures 11 mm

A revision ACLR after a re-rupture is challenging. Factors that influence the decision-making are multifactorial and involve previous tunnel position, tunnel diameter, revision graft choice, fixation method and surgeons’ own experience [32]. Two-stage techniques are less optimal due to increased convalescence of two separate staged procedures, longer overall rehabilitation time and return to activity. The main challenges in revision ACLR are due to the need to manage bone loss or damages as a result of malpositioned tunnels, or properly positioned tunnels that have expanded significantly beyond their original diameter [4]. Since the implants used are bioabsorbable and osteoconductive, one would argue that a revision surgery may prove to be less challenging if drill canals are not misplaced. Both patients who suffered a re-rupture received a 2-stage ACLR even though they had anatomically placed drill canals. Computer tomography (CT) for both patients did not reveal tunnel widening. However, the outside femoral canal diameter from the original operation is relatively wide (Fig. 5). Thus, a more conventional ACLR with an endobutton cannot be performed as a 1-stage procedure. The outside canal has a 4 mm wider diameter than the transplant canal. Experience with a revision ACLR using the T-lock method does not exist in the medical literature. As experience with the one-stage revision of the T-Lock Osteotrans was lacking and it was not clear whether fixation close to the joint with a screw or as a bony press-fit procedure was possible, a two-stage revision was considered and carried out in both patients as the safer procedure. Perhaps a study with a bigger group of patients that include several re-ruptures could provide more data on revision surgeries and whether a 1-stage procedure could be feasible.

The MRI evaluation 1 year and in some patients till 3 year postoperatively can be relatively short since evolving biologic activity and graft incorporation continues well beyond 1 year following ACLR [14]. Yet graft maturation was very good with only 1 patient showing a hyperintense signal on the MRI which suggests an incomplete graft maturation [8]. In patients with follow-up MRIs, no tunnel enlargement was seen compared to the 1-year result. A relevant enlargement would be however over 15 mm. This was not seen in any patient. Since no deformities or fractures of the anchoring device was seen, it is safe to assume that the T-lock fixation is stable 25 months postoperatively. MRI visibility seems to be improved in this case. However, metal debris from the procedure could still cause visual limitations due to metal induced artifacts.

Limitations in this study include the small patient group and the wide age range of the patients as well as big differences in the patients’ preoperative activity level according to Tegner activity score (4–10 points). The present study also reports surgical results of a new method which was done by 3 different surgeons. This may have affected the results. Furthermore, not all MRI were conducted using the same MRI scanner.

## Conclusion

ACLR with femoral fixation of the semitendinosus autograft using the T-Lock fixation method leads to good clinical and radiological outcome. It offers the advantages of near joint fixation and allows for a good return to sports rate. The bioabsorbable material enables more MRI visibility and eases revision surgery since no hardware removal is necessary. However, it is still not known if a 1-stage reconstruction procedure following a re-rupture is feasible in these patients. More studies are required with a bigger patient groups and longer follow-up durations.

## Data Availability

Data and materials used in this study can be made available upon reasonable request.

## Abbreviations

ACL: Anterior cruciate ligament
ACLR: Anterior cruciate ligament reconstruction
MRI: Magnetic Resonance Imaging
BME: Bone-Marrow-Edema
